# Barriers to successful dichoptic treatment for amblyopia in young children

**DOI:** 10.1007/s00417-021-05193-1

**Published:** 2021-05-31

**Authors:** Aveen Kadhum, Emily T. C. Tan, Dennis M. Levi, Linda Colpa, Maria Fronius, Huibert J. Simonsz, Sjoukje E. Loudon

**Affiliations:** 1 Department of Ophthalmology, Erasmus Medical Center, Room Ee 1663, P.O. Box 2040, 3000 CA Rotterdam, The Netherlands; 2 School of Optometry, Graduate Group in Vision Science and Helen Wills Neuroscience Institute, University of California, Berkeley, Berkeley, CA USA; 3 Program in Neuroscience and Mental Health, The Hospital for Sick Children, Toronto, Canada; 4 Department of Ophthalmology, Child Vision Research Unit, Goethe University, Frankfurt am Main, Germany

**Keywords:** Amblyopia, Dichoptic treatment, Barriers to successful treatment

## Abstract

**Purpose:**

In an ongoing randomised clinical trial comparing dichoptic VR video games with patching for amblyopia, we evaluated any potential barriers to successful use of this novel amblyopia treatment method.

**Methods:**

From December 2017, all newly diagnosed amblyopic children were recruited. Excluded were children under age 4 and patients with strabismus exceeding 30PD. The video game was played for 1 h per week at the outpatient clinic under direct supervision. Records were kept of difficulties encountered during treatment and categorised into domains. Factors influencing the successful completion of this treatment were identified and related to patient characteristics.

**Results:**

Ninety-one children were recruited for the trial, 20 parents refused participation before randomisation, because of the logistical challenges the outpatient dichoptic treatment would cause them. Of the 17 children who commenced dichoptic treatment (median age 6.2 years; IQR 4.9–8.4 years), 10 did not complete treatment. Children under age 5.5 years were unable to comprehend the game settings or the game itself. Older children (*N* = 7; 41%) were less willing to comply with the video game. Loss of interest in the game (*N* = 8; 47%) was found to be a limiting factor at all ages.

**Conclusion:**

Half of the children failed to complete VR dichoptic treatment, mainly due to young age. In countries with nationwide screening where amblyopia is detected before age 6, the applicability of such dichoptic treatment is limited. 
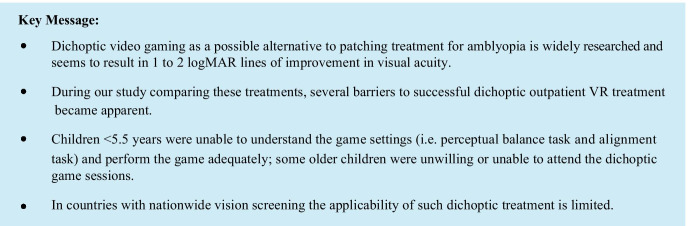

## Introduction

The past decade has seen a rise in the use of dichoptic training [1, 2] as a possible alternative or supplement to the standard patching therapy for amblyopia [3, 4]. The new dichoptic therapies are often presented as video games: stimulating the brain by repeating a set of simple tasks [5]. They are based on the theory that amblyopia is an intrinsically binocular problem: disruption of binocular vision in early childhood leads to amblyopia with suppression of the amblyopic eye [1]. Since playing a video game is expected to be enjoyable for children, it was assumed that this approach would be less of a burden for the child than patching. In addition, it has been suggested that these therapies may be more effective in improving stereoacuity [6, 7] and contrast sensitivity [8].

A number of studies have reported favourable results not only in children but also in adults with on average 1 to 2 logMAR lines of improvement [9, 10]. However, studies comparing the effectiveness of behavioural training with patching were incomplete because the actual gaming time was compared to prescribed or reported patching time [11–13]. Patching times noted by parents are often overestimated whereas compliance with patching measured electronically is poor (on average 50%), making a valid comparison difficult [3, 14]. In addition, studies of dichoptic treatment often compare 1 h of gaming to 1 h of patching. However, the treatment efficiency of gaming is reported to be higher than patching: 100–120 h of patching for each line of visual acuity (VA) gain in young amblyopes [3], and more than 200 h in older than 7 year olds [15] seems to be equivalent to 10–20 h of gaming therapy [2, 16–18]. This encouraged us to design the first trial (NCT03767985) in which we compare the effectiveness of dichoptic video gaming with electronically monitored patching therapy for amblyopia. For this study we recruited children newly diagnosed with amblyopia. A dichoptic action video game (1 h/week) using virtual reality (VR) goggles was played under direct supervision of the researcher at an outpatient clinic in the Netherlands. During the trial, it quickly became apparent that this treatment method brought along several unexpected challenges. Thus, in this report, our main focus was to present our experiences working with dichoptic action video gaming as an amblyopia treatment for children; the patching group is not discussed in this report and overall results of the randomised clinical trial (RCT) will be presented elsewhere. We present the first report describing our experiences with this new game therapy and its feasibility in orthoptic practice.

## Materials and methods

For the RCT (NCT03767985), children were recruited from four clinics between December 2017 and April 2020. The majority of the participants were from The Hague, which consists of a multi-ethnic and -cultural population with 45% being of Dutch origin and 55% of non-Dutch origin. The treating orthoptist in the clinic referred the child with newly diagnosed amblyopia to the research centre. The research orthoptist examined the child according to the study protocol, using the crowded tumbling E-chart. Amblyopia was defined as a difference in best-corrected visual acuity of 2 or more logMAR lines caused by refractive error, strabismus or a combination of the two. The decision to include the child was made by the research orthoptist, following the inclusion and exclusion criteria of the study protocol.

Based on the literature [11, 13], an age range of 4–12 years was applied. Exclusion criteria were previous treatment for amblyopia, strabismus angle more than 30PD, neurological disorder, other eye disorders and diminished acuity due to medication, brain damage or trauma. Cycloplegic refraction was performed using 1% cyclopentolate. In our study, all children who required spectacles first underwent a 16-week refractive adaptation period according to a standardised protocol. This was a prerequisite for the study. If visual acuity difference was less than 2 logMAR lines after refractive adaptation, hence not meeting the criteria for amblyopia, they were not eligible for randomisation. Other parameters, i.e. age, gender, diagnosis, were also documented.

The Ethics Committee of Erasmus University Rotterdam and the Boards of the participating clinics approved the protocol and informed consent forms. Written informed consent was obtained from each subject and/or from his or her parents or guardians. The research adhered to the tenets of the Declaration of Helsinki.

We kept records of all difficulties encountered during the study and created a diagram representing the factors that influenced the success of dichoptic game treatment. We created a focus group, consisting of the research team, two independent orthoptists and two paediatric ophthalmologists as experts in the field. During multiple sessions with our focus group, these factors were discussed, evaluated and categorised into three domains: (1) equipment and usage, (2) child and parental adherence with therapy and appointments and (3) costs. All factors weighed equally and were systematically scored per child; each domain will be discussed separately below.

### Equipment and usage

#### Hardware

**Fig. 1:**
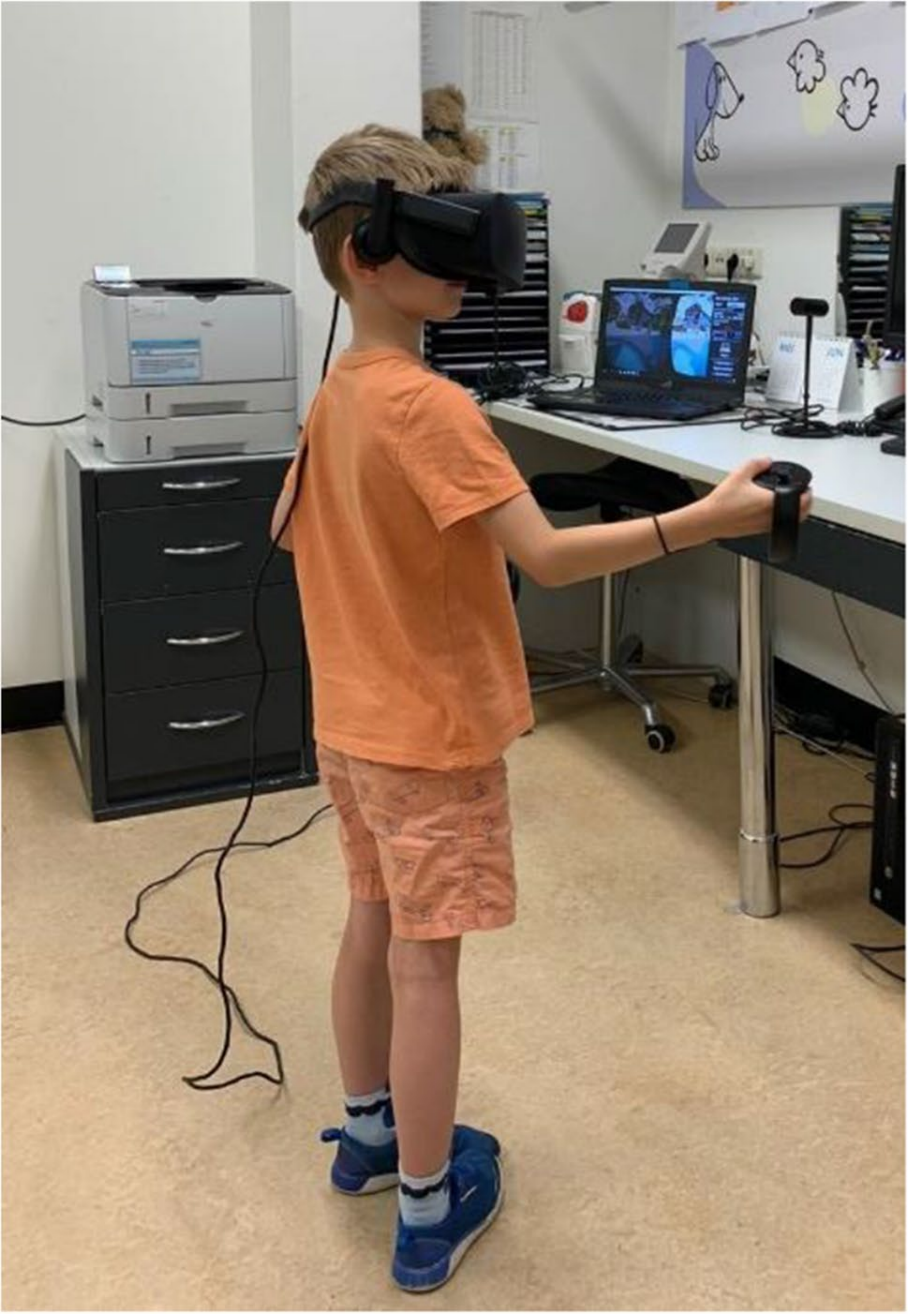
A 6-year-old boy playing the game. He is wearing the VR goggles and using the controllers to play the game. The laptop on the desk shows the split screen with the left eye being the fellow eye and hence displaying a reduced contrast/luminance

The devices used to perform the dichoptic game were the Oculus Rift and the laptop Asus ROG Strix SCAR Edition GL503VS-EI012T. This was a fixed set-up located at the outpatient clinic (Fig. 1).

#### The game

The software included an active and engaging game for children with settings for perceptually balancing the images seen by the two eyes (by attenuating the contrast/luminance of the image seen by the dominant eye), and the ability to correct for alignment at the start of each game session. The video game was custom-made, based on the principles of the previously reported dichoptic games developed by Levi et al. [4, 17] and modified by Alting (Dfab). The game consisted of two different game surroundings (marketplace and cave), with difficulty increasing during game play. The child, wearing the VR goggles and holding the controllers, was standing in the marketplace. Snowmen appeared and the child was instructed to throw snowballs at the approaching snowmen. Points were awarded for hitting the snowmen. A suppression check was incorporated in the form of a snowflake, which was presented every 30 s for 10 s solely to the amblyopic eye. The child was instructed to catch the snowflake before it disappeared to gain extra points. More importantly, successfully catching the snowflake would confirm that the amblyopic eye was still engaged.

**Fig. 2:**
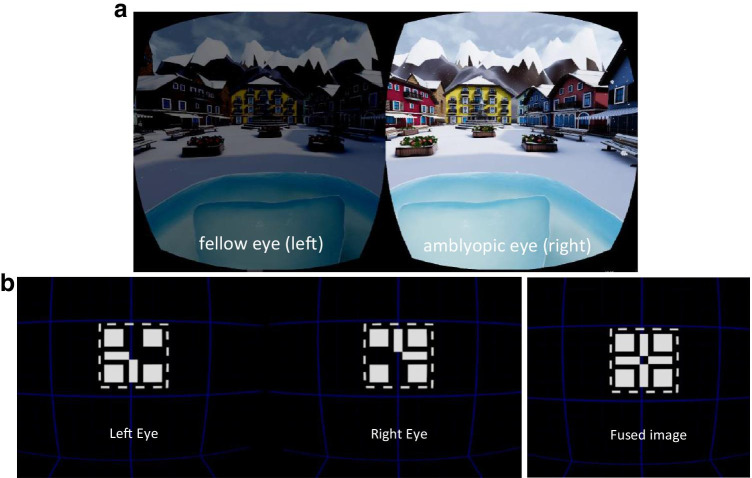
**a** Dichoptic presentation with attenuated contrast/luminance for the fellow eye (left eye) in order to match the image perceived by the amblyopic eye (right eye). **b** Alignment task with two nonius lines to fuse into one full cross. The image on the right shows the full cross perceived when the two images are fused

Prior to each game session, a perceptual balance and alignment task was performed. Firstly, for the perceptual balance task, two images were presented dichoptically and the contrast/luminance presented to the fellow eye was modulated in order to match the appearance of the high-contrast image perceived by the amblyopic eye. The researcher adjusted the contrast/luminance based on the feedback of the child. The task was repeated four times and the mean contrast/luminance level was applied (Fig. 2a). Balancing the perceptual input to the two eyes is purported in the literature to reduce suppression and is believed to be a key factor in dichoptic therapy effects on visual acuity and stereoacuity [1, 19]. The primary goal of the perceptual balance task was to reduce suppression and facilitate fusion. We chose to base the level of contrast/luminance subjectively on the patient’s feedback as opposed to randomly assigning a contrast level to ensure genuine conditions.

Secondly, the alignment task was performed according to the principles used in previous studies in both adults and children [4, 17, 20]. This included the presentation of two nonius lines dichoptically (Fig. 2b). These two images had to be aligned properly until a full cross was perceived. Both the perceptual balance and alignment tasks were based on the patient’s subjective responses.

### Child and parental adherence with therapy and appointments

Children who did not bring their spectacles to a game session had to be rescheduled.

Dichoptic gaming treatment in our study was conducted once a week at the outpatient clinic and comprised a total of 24 sessions. This meant weekly trips to the clinic by the patient with at least one parent or supervisor. Each game session commenced with the perceptual balance and alignment settings followed by 1 h of game play with breaks in between. All sessions were directly supervised by the researcher enabling objective monitoring of compliance. Compliance during each game session was recorded with a stopwatch. Compliance with the scheduled weekly game session appointments during the total therapy duration was also recorded.

### Costs

We assessed all costs involved for the health care provider as well as the patient. This included the following: equipment, software and maintenance/updates, personnel supervising the game sessions, treatment room rent, overhead and travel costs.

## Results

**Fig. 3:**
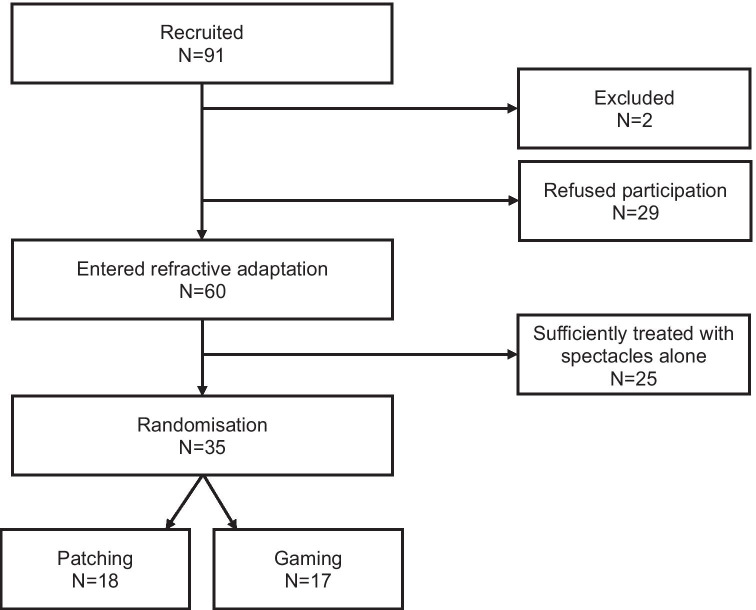
Flowchart with recruitment of children for the randomised controlled trial

For the RCT, 91 children (age 4–12 years) were recruited by the treating orthoptists; all records were analysed (Fig. 3). Two children were excluded based on linguistic problems and legal issues. The parents of 29 children refused participation before randomisation, 20 for reasons directly related to the dichoptic game treatment: 18 were unwilling or unable to comply with the weekly game sessions, one parent refused participation as he thought the game treatment would be harmful for his child’s eyes and one child was frightened by the prospect of the game. After the refractive adaptation period, amblyopia was sufficiently treated in 25 children, i.e. visual acuity difference between both eyes resolved to less than 2 logMAR lines. Thirty-five were randomised into the two arms of the study: 18 to patching and 17 to the dichoptic gaming group. The 17 children assigned to the game group were included in this study and are the subject of this paper.

### Visual acuity

No children were excluded based on their visual acuity as it did not limit the ability to conduct the treatment. No apparent relationship could be found between visual acuity in the amblyopic eye at start of treatment and the ability to perform the dichoptic game treatment.

### Strabismus

Based on the literature, patients with a strabismus angle > 30PD were excluded [4]. In our study in the gaming group, there were only two subjects with strabismus: the first patient had 10PD partial accommodative esotropia, hypermetropia with dubious binocular single vision; the second patient had 12PD fully accommodative esotropia, hypermetropia with demonstrable binocular single vision. These subjects with strabismus did not complete the game treatment; however, their angle of strabismus was not the main reason for them being unable to conduct the therapy.

### Age

Median age of the gaming group was 6.2 years (IQR 4.9–8.4 years). Median age of those who dropped out was younger, 5.4 years (IQR 4.8–7.3) compared to 6.7 years (IQR 5.4–12.3) in the children who completed the game treatment; however, this was not significant (*P* = 0.27; Mann–Whitney *U* Test).

### Equipment and usage

#### Hardware

Initially, the dichoptic video game was played using Zeiss 3D OLED goggles. In practice, we experienced difficulty fitting the subjects’ own spectacles underneath these 3D goggles. Moreover, during game play, there was no external screen for the researcher to verify the image seen in the OLED goggles by the child, therefore making it impossible to track the game progress during game sessions. To correct these obstacles, we changed to the Oculus Rift VR goggles (see Fig. 1).

**Fig. 4:**
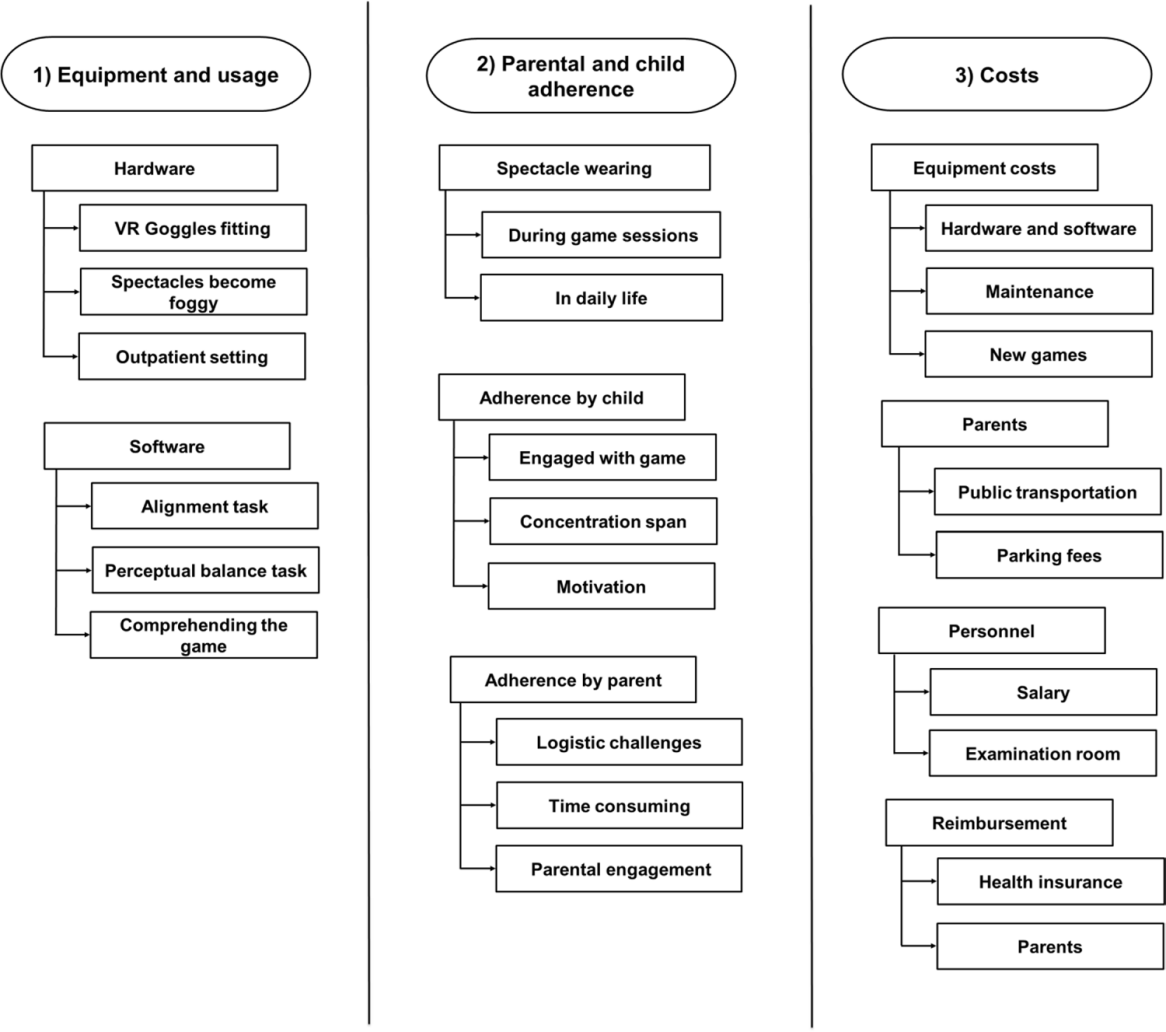
Overview of the challenges for each domain

The laptop together with the Oculus Rift had to be set up adequately for the space where the game sessions were conducted. This set-up was intended for use at the outpatient clinic and was not easy to transport as it was bulky and heavy (Fig. 4).

The Oculus Rift was more appropriate for older children due to the size and weight of the headset and controllers. However, in 24% the spectacles would become foggy underneath the headset during active game play, resulting in a blurry image. If children reported this, the game session was interrupted in order to clean the spectacles. Foggy spectacles could not be directly observed by the supervisor, so it is possible that this occurred more often than was reported. Keeping the spectacles clean was essential as presenting a clear and sharp image to both eyes during game play was a critical element of the therapy and had to be maintained at all times. These breaks led to frustration by the child and loss of concentration.

#### The game

As depicted in Table 1, children younger than 5.5 years had difficulties applying the game settings, i.e. they did not understand the perceptual balance task and/or could not communicate properly whether they perceived a full cross with the alignment setting. In addition, these children were also unable to comprehend the task of throwing snowballs at the approaching snowmen and would often just look around in the VR goggles. Overall, 7 children did not complete the treatment due to difficulties with the game settings.

**Table 1:** This table shows the scoring sheet used to tabulate the challenges for each child. The sheet is arranged according to age. Some patients had more than one challenge. Seven children completed the dichoptic treatment. T time of drop-out in weeks during the trial. T0 refers to drop-out during the first game session trial, T2 refers to drop-out after 2 game sessions (2 weeks) and T6 after 6 game sessions (6 weeks)

Patient	Time of drop-out	Age	Difficulties with game settings	Difficulty comprehending the game	Lost interest in the game	VR goggles and controllers too large	Foggy glasses	Forgot to take spectacles to game session	Unable/unwilling to attend game appointments
No 16		4.06	X	X	X	X			X
No 1	T0	4.51	X			X			
No 5	T0	4.67	X	X	X	X			
No 15	T0	4.87	X	X					
No 9	T2	5.00			X	X			
No 14	T6	5.34	X	X	X	X			X
No 3	T0	5.37	X						
No 8		5.41			X			X	
No 11		6.16			X		X	X	X
No 2	T0	6.27	X						
No 13		6.66				X	X		
No 4	T6	6.67			X	X			X
No 10		7.41				X			
No 12	T0	9.37							X
No 17	T0	10.55							X
No 7		12.33			X		X		X
No 6		12.46					X		
Total			7/17 = 41%	4/17 = 24%	8/17 = 47%	8/17 = 47%	4/17 = 24%	2/17 = 12%	7/17 = 41%

### Child and parental adherence with therapy and appointments

Children who refused to wear their spectacles were excluded from participating, as this would preclude optimal treatment. In addition, optimal spectacle correction is essential for obtaining or improving binocular vision; for example, a patient with a fully accommodative esotropia. Two eligible children were excluded due to refusal to wear spectacles.

During the trial, on occasion some children would forget to bring their spectacles. Children who showed up at the appointment without their refractive correction had to be rescheduled. This occurred in 3% of the scheduled appointments.

Table 1 shows that boredom with the game was apparent in the younger, but also in older children. During the lengthy sessions or whenever they lost interest in the game, they would simply stop throwing snowballs and refuse to continue. In our study, each game session lasted a minimum of 1.5 h: one h for gaming and 30 min for doing the settings and breaks in between. On consecutive appointments, the child would become increasingly reluctant to come in and play the game.

Table 1 shows that approximately half of the children (41%) were unwilling or unable to comply with the weekly game sessions. Parents had to implement the weekly 1.5-h game session into their schedule; this excluded travel time to and from the clinic. For this reason, 18 eligible subjects refused participation a priori, because they found the game treatment too much of a burden and difficult to incorporate into their daily life. For trial participants, within the gaming group there were 3 children who dropped out due to these same logistical challenges. In addition, many families had both working parents and siblings having other commitments (e.g. sports), which often resulted in limited time to attend the clinic. Another type of non-compliance was found during the study: parents of children in the game group frequently cancelled their appointments often mentioning that their child was not interested in playing the game anymore. Overall adherence was not related to age.

### Costs

Several costs were identified (Fig. 4). Firstly, the required *hardware* to perform the game including the laptop and virtual reality headset. Secondly, the *software*: the development and modifications of an engaging child-friendly dichoptic video game with two different game environments, including settings for perceptual balance and alignment and a suppression check. In addition, in our study, the game sessions were conducted under direct supervision once a week at the outpatient clinic. This resulted in personnel costs: an orthoptist needed to supervise the game session. Then, there are the travel expenses, parking fees and the cost of time off work for the parents to be taken into account.

## Discussion

We recorded factors that influenced the applicability of dichoptic video gaming with VR goggles in young children. These factors ranged from recruitment of an eligible patient up until successful completion of dichoptic treatment. Almost all parents who refused to participate prior to inclusion were unable or unwilling to engage in outpatient dichoptic treatment; and half of the included children did not complete the treatment. Overall, we found that children younger than 5.5 years of age had too much difficulty with the game settings, difficulty comprehending the game and 1 h of active gaming was too tedious for them. Older children (and their parents) were unwilling to adhere to the weekly game schedule. Losing interest in the game was apparent at all ages.

Age turned out to be a key factor in determining eligibility and success of dichoptic treatment in practice. In the literature, the age of children undergoing these therapies mostly range from 4 to 17 years [10]. We note that the subjects from the study of Gambacorta et al., which used the same gaming principles, had older subjects with an age range of 7 to 17 years [17]. We found that young children, with limited language skills and cognitive ability, had more difficulty comprehending the game as well as understanding and articulating feedback concerning for example the perceptual balance and alignment settings. In countries such as the Netherlands, with an extensive vision screening program, children with strabismic amblyopia are detected at 2.5 years of age and those with refractive amblyopia at 4.5 years of age [21]. This raises the question as to whether this type of therapy would be feasible. Children with a large strabismus angle (> 30PD) were excluded; we only had two children with a strabismus angle up to 12PD who were able to fuse the images. The reason for these children to drop out was their inability to fully understand the game settings (ocular alignment and contrast settings). However, one could hypothesise that the second child especially, based on her small-angle strabismus and some degree of demonstrable binocular single vision, would have been able to conduct the game. From clinical experience, we would expect children with a larger strabismus angle to have more difficulty fusing the images. Several studies based on dichoptic iPad treatment using anaglyphic glasses applied an even smaller strabismus angle as exclusion criteria, excluding all patients with deviations ≥ 10PDor even ≥ 4PD [11, 18]. As children with strabismic amblyopia are detected at an earlier age, treatment should commence as soon as possible rendering them ineligible, not only because of the angle of strabismus but also their age. This would indicate dichoptic treatment in children would be at best feasible for small-angle strabismic/combined amblyopes or anisometropic amblyopes that are first diagnosed at an older age, or in countries with less successful early detection and treatment programs for amblyopia.

In our study, the game was played under direct supervision of the researcher. This design was chosen to ensure the game therapy was conducted correctly and to monitor compliance. However, this set-up revealed its own challenges. Due to a fixed game set-up at the outpatient clinic, parents had to incorporate this into their daily routine and maybe even take time off work—the costs of which needs to be considered by all parties. Ideally, a home-based alternative would be offered; however, Holmes et al. reported poor compliance with iPad games at home [11, 12]. In addition, moving to a home-based setting would require more parental responsibility and supervision to ensure the sessions are performed correctly and, with VR, avoiding injury if children move around with the goggles on.

Patient motivation with the game therapy is essential. Unlike adults with amblyopia, who are generally intrinsically motivated to improve their eyesight and therefore to comply with treatment, children have to be kept engaged. Young children, especially, have more difficulty comprehending the reasons for treatment. Moreover, these young children in general have a shorter concentration span and get more easily distracted during the game. Therefore, games should be aimed at keeping children engaged according to their age group. Young children need a gaming environment with minimal stimuli and simplistic objects; older children need a more complex and varied gaming environment with more stimuli to keep them engaged. Ideally, there should be a variety of different highly engaging games with rich environments for different age categories. This would come with high costs. Important to note is that the video game industry is a whole separate branch developing rapidly with large teams set up specifically to develop games. Games developed by research groups cannot match the quality of games developed by the industry, due to their expertise and experience, so ideally researchers should work together with the game industry to produce compelling video games. However, regardless of offering a broad range of games suitable for different age categories, we cannot overlook the psychological factor that assigning a child to play a video game as a therapy is not the same as when a child voluntarily chooses to play a game; therefore, compliance rates should not be overestimated.

The costs of conducting dichoptic treatment with VR goggles were considerable. This raises the question who will pay for these costs: the national or private health insurance, or out of pocket of the families. Our set-up in the clinic made it labour intensive and therefore more expensive.

The VR goggles used in this game therapy were not primarily designed for young children. New inexpensive consumer VR headsets such as the Oculus Go, that can be operated via a cell phone, may help to offset some of these issues. Offering dichoptic therapy for amblyopia in other forms, such as using an iPad or dichoptic movie watching, may be more suitable for younger children [22]. Nevertheless, there were several other aspects limiting the success of this type of treatment that would still be present with these alternative forms, such as issues with compliance and logistics.

As awareness of these new therapies rises, this has its effect on daily orthoptic practice. With this inventory we hope to provide treating orthoptists guidelines for informing parents about these new treatment methods.

## Data Availability

Not applicable.
